# Mutual Benefit Association

**Published:** 1883-12

**Authors:** 


### Mutual Benefit Association.

An organization having the name of "*The Dentists' Mutual Benefit Association*" has recently been organized at Kansas City, Mo. The object and purposes of the society are conveyed by the name. Similar organizations have been established in many of the avocations of life, and when properly conducted accomplish great good, as by this means every member is enabled to make some provision for those dependent upon him, which is always a desirable thing.

We are often reminded of the fact that men in our own profession are taken away without any provision for the support of their families. Many notable cases of this kind have occurred in the dental profession within the last few years, and some of them, at least, because of their intense and all-absorbing devotion to their profession.

This Association secures for all its members immunity, in a measure at least, from such disaster.

In section 3, article 1, of the constitution, it is stated that "The objects of this Association are to increase the interest in the science of dentistry, to facilitate and secure a more extended acquaintance among the members of the dental profession, and to provide pecuniary aid for the widows, orphans, heirs, and devisees of deceased members of said Association."

Section 1 of article 2 says: "Any man of good moral character, and in good general health, and not over fifty years of age, who is now and has been for one year immediately prior to the date of his application, engaged in the active practice of dentistry, and a member in good standing, either active or honorary, of some regularly local, State, or district dental society or association, within the United States, is eligible to membership in this Association."

The President is Dr. C. H. Darly, of St. Joseph, Missouri, and Dr. R. J. Pearson, of Kansas City, Missouri, is Secretary and Treasurer.

The Advisory Board consists of well-known members of the profession of Missouri, Kansas, Illinois, Ohio, New York, Pennsylvania, Massachusetts, Georgia, Texas, Iowa.

The constitution, by-laws, and rules, together with any other information, can be obtained of Dr. R. J. Pearson, of Kansas City, Missouri.

